# Clinical outcomes associated with drug–drug interactions involving buprenorphine: a systematic review

**DOI:** 10.1186/s13722-026-00698-8

**Published:** 2026-07-09

**Authors:** Jude Uche Eze, Easter Protiva Gain, Nistha Shrestha, Yu-Jung Jenny Wei

**Affiliations:** Division of Pharmaceutics and Pharmacology, College of Pharmacy, The Ohio State University, 414 Riffe Building, 500 West 12th Avenue, Columbus, OH 43210 USA

**Keywords:** Buprenorphine, Drug-drug interactions, Clinical outcomes

## Abstract

**Background:**

Increases in prescribing of buprenorphine for pain or opioid use disorder (OUD) have led to concerns regarding its interactions with other drugs used concomitantly and risk of adverse clinical outcomes.

**Objective:**

To synthesize current evidence on associations between buprenorphine–drug interactions and clinical outcomes among patients with pain or OUD.

**Method:**

A systematic review was conducted by searching MEDLINE, CINAHL, APA PsycINFO, and Web of Science from inception through June 2025 on studies examining buprenorphine–drug interactions and clinical outcomes. The PRISMA 2020 guideline was used for assessing and reporting data quality. Reviews were included if they reported clinical outcomes such as OUD, opioid overdose (OD), all-cause mortality, or emergency department (ED) visits. Data extraction and risk of bias assessment were performed independently by 2 reviewers. Heterogeneity of interacting drug exposure and outcomes studied precluded meta-analysis.

**Results:**

Seven studies were included. All studies were observational in design, focused on patients with OUD, and evaluated only pharmacodynamic, not pharmacokinetic, interactions involving buprenorphine with central nervous system depressants. The reviewed studies showed an increased risk of OD in patients who co-used buprenorphine with benzodiazepine, Z-drugs, pregabalin, gabapentin, clonidine, or oxycodone (vs. buprenorphine only), with mixed results for non-fatal OD being noted in concomitant buprenorphine-benzodiazepine use. An increased risk of drug-related poisoning (including opioid overdose) was observed in patients with co-use of buprenorphine and benzodiazepine, with null results being noted in patients with co-use of buprenorphine with gabapentin or prescription amphetamines (vs. buprenorphine only). An increased risk of all-cause mortality was observed in patients with co-use of buprenorphine with Z-drugs or pregabalin, with mixed results being noted in concomitant buprenorphine-benzodiazepine use (vs. buprenorphine only). Only one study showed an increased risk of all-cause ED visits in patients with buprenorphine-benzodiazepine use vs. with buprenorphine alone.

**Conclusion:**

This review suggests inconclusive evidence on potential adverse outcomes associated with concomitant buprenorphine–drug use compared with buprenorphine use only. Further population-based studies with rigorous study designs are needed to elucidate associations between buprenorphine–drug interactions and clinical outcomes.

**Registration:**

PROSPERO CRD420251052355.

**Supplementary Information:**

The online version contains supplementary material available at https://doi.org/10.1186/s13722-026-00698-8.

## Introduction

Buprenorphine is an opioid medication approved by the U.S. Food and Drug Administration (FDA) to treat both pain and opioid use disorder (OUD), although most buprenorphine prescribing has been for treatment of OUD [1, 2] When used to manage pain, buprenorphine transdermal and buccal forms are available, with the dose being prescribed in micrograms. When used to manage OUD, buprenorphine sublingual and buccal forms are used, with the dose prescribed in milligrams [3]. Use of buprenorphine has increased substantially over the past decade. Between 2009 and 2018, prescribing for buprenorphine indicated for OUD rose from 1.97 per 1,000 population to 4.43 per 1,000 population [4]. More recent data from the Centers for Disease Control and Prevention indicate that national buprenorphine dispensing remained relatively stable from 2019 to 2023, with 4.7 prescriptions dispensed per 100 persons in 2023, totaling more than 15 million prescriptions nationwide [5].

The continued growth of buprenorphine use is largely attributed to its partial µ-opioid receptor agonist activity and ceiling effect at higher doses, which limit the risk of respiratory depression and overdose compared with full opioid agonists. Moreover, buprenorphine seems to carry a lower risk of misuse and diversion than methadone and other full-agonist opioids [6], although concern has been raised about potential misuse of buprenorphine without a prescription or following a physician’s instruction [7]. Because of its favorable safety profile and benefits in decreasing opioid cravings and withdrawal symptoms and reducing risk of overdose-related mortality, buprenorphine is considered a cornerstone of OUD treatment and has been increasingly recommended by clinical experts for managing both non-cancer and cancer-related pain [4, 8, 9].

With increasing prescribing of buprenorphine for patients with OUD or pain, clinical concern has been raised about interactions and the subsequent risk of clinical adverse outcomes in real-world settings when individuals receive buprenorphine concomitantly with other drugs. Buprenorphine can interact with other drugs in two ways, including pharmacokinetic (PK) interactions that involve changes in drug concentration, and pharmacodynamic (PD) interactions that result from opposing, additive, or synergistic drug effects on the central nervous system (CNS) [1, 10–13]. In PK interactions, because buprenorphine is mainly metabolized via the enzyme cytochrome P450 3A4 (CYP3A4), co-use of buprenorphine with drugs that inhibit the CYP3A4 enzyme, such as azole antifungals (e.g., voriconazole) or macrolide antibiotics (e.g., clarithromycin), can reduce buprenorphine metabolism and increase its blood concentration. This increase could heighten the risk of respiratory depression and opioid overdose [2, 12, 13]. Co-use of buprenorphine with drugs that induce the CYP3A4 enzyme, such as rifampin or certain anticonvulsants (e.g., carbamazepine), can reduce buprenorphine concentration, leading to reduced analgesic effects and uncontrolled pain as well as triggering precipitated opioid withdrawal symptoms (e.g., agitation) and opioid cravings, particularly in individuals with OUD [14]. In PD interactions, co-use of buprenorphine with CNS depressants (e.g., benzodiazepines) potentially increases sedation, leading to risk of fall injuries, CNS respiratory depression, and opioid overdose [15, 16]. Indeed, of 3,241 buprenorphine-involved overdose deaths in the U.S. between 2010 and 2017, 46.9% (95% CI 43.6–50.2) involved benzodiazepines, and 17.8% (95% CI 15.3–20.5) involved alcohol [17]. In 2017, the U.S. Food and Drug Administration issued a warning against concomitant use of medications for treating OUD, including buprenorphine, with benzodiazepines and other CNS depressants [18].

An increasing number of observational studies have been conducted to examine clinical adverse outcomes associated with drug–drug interactions involving buprenorphine in real-world settings. To inform clinical prescribing of buprenorphine with other drug therapy, this review study aims to evaluate current evidence on associations between buprenorphine–drug interactions and risk of clinical outcomes for patients with pain or OUD assessed in randomized clinical trials or observational studies.

## Methods

A comprehensive literature search was conducted in MEDLINE, CINAHL, APA PsycINFO, and Web of Science from inception through June 2025 (detailed in Appendix S1). We used a pre-specified protocol (PROSPERO CRD420251052355) following the Preferred Reporting Items for Systematic Reviews and Meta-Analysis (PRISMA) 2020 guidelines. This study received an exemption from review from the Ohio State University’s Institutional Review Board because the study used published data and did not involve human participants or identifiable patient information.

### Search strategy and selection criteria

We combined MeSH and free-text keywords related to buprenorphine, OUD, pain management, drug–drug interactions, and clinical outcomes, using Boolean operators and field-restricted queries (e.g., TX, MH, AB, TI). Eligible studies included randomized clinical trials and observational designs of cohort, case-control, case-crossover, or self-controlled case series studies, but not cross-sectional studies or case reports. Populations were adults ≥ 18 years receiving buprenorphine for OUD or pain. Exposures were concurrent use of buprenorphine with CNS depressants or drugs that inhibit or induce CYP3A4. Comparators included buprenorphine alone, placebo, or co-use with a drug that did not appear to be associated with the clinical adverse outcomes of interest. Studied outcomes included opioid poisoning, drug-related poisoning, opioid overdose, opioid withdrawal symptoms, respiratory depression, CNS depression, hospitalization, emergency department (ED) visits, adverse drug reaction, harm, or death (Appendix S2). Drug-related poisoning encompasses poisoning by opioids, benzodiazepines and sedatives, psychotropic agents, CNS stimulants, cardiovascular agents, and other substances, and therefore includes but is not limited to opioid overdose or poisoning. Only English-language human studies were included (Appendix S3).

### Data management

We uploaded references identified using our search strategy to Covidence, a web-based collaboration software platform for managing duplicate publications and standardizing our literature reviews [19]. Two investigators (J.U.E. and E.P.G.) independently reviewed titles and abstracts of all identified studies as well as full-text articles that were considered potentially eligible. Disagreements were resolved through consensus and, when necessary, by a third independent reviewer (N.S.) and senior investigator (Y.J.W.).

### Data extraction

We developed a standardized data extraction form for this review, informed by the structure and recommendations provided by the Covidence systematic review software [18]. The form was used to extract study characteristics and key findings from each included article and its supplementary materials [20]. Extracted items contained characteristics and key findings of the reviewed articles. Study characteristics included study identification (author and year), study design, data source, country and clinical setting, study population, and key patient demographics (age and sex), co-occurring diagnosis, and indication for buprenorphine use. Key findings included study groups (co-use of buprenorphine with an interacting drug), comparator groups (placebo, buprenorphine only, or co-use of buprenorphine with a drug considered as an active comparator for the studied interacting drug), type of drug–drug interaction (PD or PK) and its exposure assessment window, outcome and its definition, source, and timeframe for ascertainment, and effect measures and associated 95% confidence intervals (CIs). Depending on the source study, we reported effect measures derived from original studies, which may include odds ratios (ORs), adjusted risk ratios (aRRs), adjusted odds ratios (aORs), or adjusted hazard ratios (aHRs). To facilitate interpretability across different effect estimates, we converted the HR and OR estimates to RR using the widely used formulas by VanderWeele [21, 22]. Given that the outcome event rate of the study and comparison group was mostly not reported in studies, we converted the HRs to RRs under the assumption that the outcome event rate is unknown but estimated between 0.2 and 0.8 for both study and comparison groups. The ORs were converted to RRs under the assumption that the outcome event rate in the comparison group is unknown, but less than 10%.

### Risk of bias assessment

Risks of bias were assessed by two independent reviewers (J.U.E. and E.P.G.), with disagreements resolved by consensus. All identified studies were observational studies. To assess the quality of cohort and case-control studies, we used the Newcastle–Ottawa Scale (NOS) and assigned stars for the standard domains of Selection, Comparability, and Outcome/Exposure, with a higher number of stars indicating a higher quality of the article [23] (Appendix S4). For case-only designs (case-crossover and self-controlled case series), we applied an adapted version of the NOS that was tailored to the methodological features of these designs [24].

### Statistical analysis

We described the characteristics of the included studies and prioritized risk of bias, indication for buprenorphine use in the study population, type of drug-drug interaction, and type of comparison group for data synthesis. We presented the study results by types of clinical outcomes assessed and type of interacting drug class. We refrained from conducting a meta-analysis of eligible studies because of heterogeneity in study designs, types of concomitant buprenorphine–drug use, and outcome measures. We attempted to consider alternative quantitative data synthesis methods (e.g., summarizing the effect estimates, combining P-values, and vote counting based on direction of effect) [25], but refrained from applying these methods because a wide range of outcomes were studied, with limited studies (*n* ≤ 2) focusing on the same outcomes of interest for data synthesis within each type of interacting drug with buprenorphine.

## Results

**Fig. 1:**
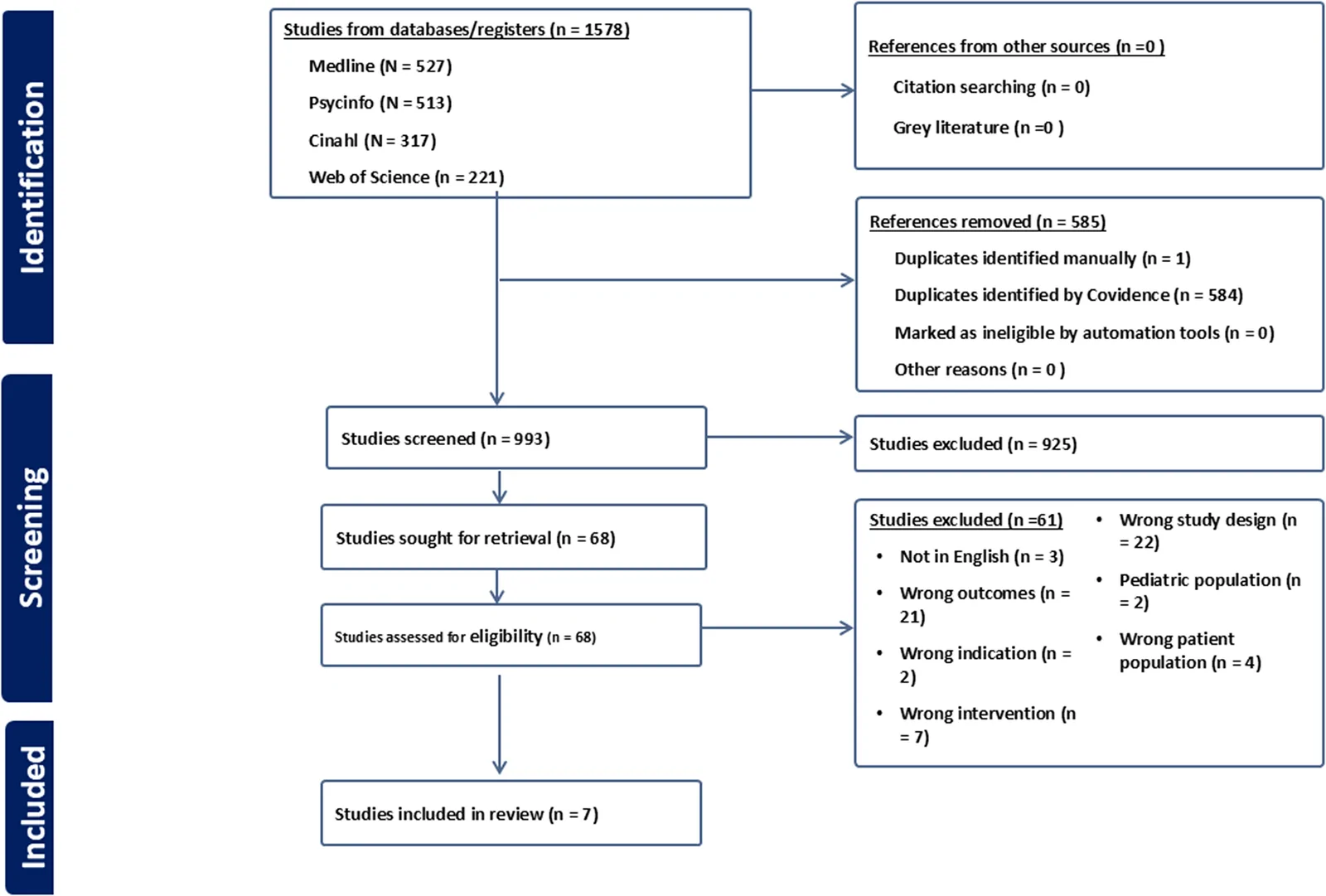
PRISMA flow diagram illustrating the study selection process

In the initial search, we included 1,578 articles through the pre-specified search strategy and then removed 585 duplicates, leaving a total of 993 articles for screening by title and abstract (Fig. 1). Of these articles, 925 were excluded as not relevant. Thus, 68 full texts were assessed for study eligibility, with 61 excluded for reasons including wrong study design (*n* = 22), wrong outcome (*n* = 21), wrong intervention (*n* = 7), wrong indication (*n* = 2), wrong patient population (*n* = 4), not in English (*n* = 3), and pediatric population (*n* = 2). Seven studies met all criteria and were included in the qualitative synthesis. All seven included studies were observational, with three studies using cohort designs and the other four using within-person designs (case-crossover or self-controlled case series) to eliminate confounders that do not change over time (Table 1) [21–27]. We did not find any RCT studies. Six of the seven included studies were conducted using U.S. data, and one study used data from Sweden. Publication years spanned 2013 to 2023, with the midpoint year of publication being 2017. The range of sample sizes in the included studies was wide, from 328 to 248,069 participants. The mean age of participants was between 30 and 40 years, and the percentage of male participants was between 50% and 74%.

**Table 1:** Characteristics of included studies (*N* = 7)

Study	Study design	Data source	Country	Study population	BUP User Status, Formulation, and Dosage	Age (years)	Sex (%)	Co-occurring diagnosis	Indication for BUP
Abrahamsson et al., 2017		National registers	Sweden	4,501 patients with opioid dependence and prescribed BUP or methadone	Prevalent BUP users, sublingual BUP, dose (NR)	Median 34.4	73.8% male	NR	OUD ^a^
Park et al., 2020	Cohort	Single state’s data warehouse	U.S.	63,389 patients with OUD diagnosis and BUP initiation	New BUP users, sublingual BUP/naloxone tablet or film, dose (NR)	Median 34	60.0% male	Chronic pain, cancer	OUD ^b^
Xu et al., 2021	Case-crossover	Commercial healthcare claims	U.S.	23,036 patients with an OUD diagnosis and ≥ 1 drug-related poisoning and prescribed BUP	Prevalent BUP users, sublingual BUP/naloxone, mean daily dose 15.4 mg (SD 7.31)	Mean 30.1	51.0% male	NR	OUD ^c^
Miano et al., 2022	Self-controlled case series	Commercial healthcare claims	U.S.	248,069 patients with opioid overdose/ADEs and BUP or methadone initiation	New BUP users, sublingual or transdermal BUP, median dose 16 mg/day	Median 60.0	50.0% male	NR	OUD ^c^
Ellis et al., 2023	Case-crossover	Commercial healthcare claims	U.S.	18,192 patients with OUD, BUP initiation, and ≥ 1 drug-related poisoning event	New users, sublingual BUP/naloxone, dose (NR)	NR	NR	Neuropathic pain, CKD, epilepsy, irritable bowel syndrome, fibromyalgia, insomnia, migraine, epilepsy	OUD ^c^
Tardelli et al., 2023	Case-crossover	Commercial healthcare claims	U.S.	8,170 patients with polysubstance use disorder, BUP initiation, and ≥ 1 drug-related poisoning event	New BUP users; sublingual BUP/naloxone; dose (NR)	NR	NR	NR	OUD ^c^
Schuman-Olivier et al., 2013	Cohort	Electronic medical records from a clinical setting	U.S.	328 patients with OUD and prescribed BUP/naloxone	Prevalent BUP users, buprenorphine/naloxone sublingual tablet, dose (NR)	Mean 35.9	60.7% male	NR	OUD ^d^

Abbreviations: ADE, adverse drug event; BUP, buprenorphine; CKD, chronic kidney disease; OUD, opioid use disorder; SUD, substance use disorder. NR, not reported

^a^ OUD was defined as having opiate dependence for at least one year according to Swedish opioid maintenance treatment regulations

^b^ OUD was identified through a filled buprenorphine prescription recorded in the Massachusetts Prescription Monitoring Program

^c^ OUD was identified as having a diagnosis of opioid use disorder coded in insurance claims data

^d^ OUD was identified through nurses’ assessments for patients who received buprenorphine in office-based opioid treatment programs

The methodological quality of the studies included was high and rated as low risk of bias (Appendix S4). All seven eligible studies included populations with OUD who received buprenorphine treatment, with no studies focusing on populations with chronic pain treated with buprenorphine (Table 1). All analyses evaluated PD interactions, focusing on the concurrent use of buprenorphine with CNS depressants or other psychotropic agents (Table 2). All studies compared patients or patient-times exposed to co-use of buprenorphine and an interacting drug with patients or patient-times exposed to use of buprenorphine only, without an active drug comparator that had a similar treatment indication but no known adverse interaction with buprenorphine (Table 2). The interacting drug classes examined were benzodiazepines (*n* = 4; 57%), Z-drugs (*n* = 2; 29%), gabapentinoids (gabapentin or pregabalin; *n* = 2; 29%), clonidine (*n* = 1; 14%), prescription amphetamines (*n* = 1; 14%), and oxycodone (*n* = 1; 14%) (Table 2). None of the studies evaluated PK interactions by assessing outcomes associated with the co-use of buprenorphine with CYP3A4 inhibitors or inducers. The clinical outcomes assessed included opioid overdose, drug-related poisoning (including opioid overdose), all-cause mortality, and ED visits (Table 3) [26–32].

**Table 2:** Summary of interaction types and measurement of drug exposure and outcomes

Author, Year	Study group	Comparison group	Type of Interaction	Exposure Window	Outcome of Interest	Source for Outcome Ascertainment	Timeframe for Outcome Measurement
Abrahamsson et al., 2017	3 study groups: Person-time with BUP plus BZD, BUP plus Z-drugs, and BUP plus pregabalin	Person-time with BUP only without the interacting drugs	PD	90 days following dispensation of BUP with concurrent prescriptions for BZD, Z-drugs, or pregabalin	Opioid overdose, all-cause mortality	Cause of death register	From July 1, 2005, to December 31, 2012.
Park et al., 2020	Person-time with BUP plus BZD	Person-time with BUP only without BZD	PD	From BUP prescription fill date to the end of the medication’s days’ supply; discontinuation defined by a ≥ 30-day gap	Opioid overdose, all-cause mortality	Mortality records, PDMP data	From the 1st BUP prescription fill as early as January 1, 2012, to December 31, 2015
Xu et al., 2021	Patient-time with BUP plus BZD/Z-drugs	Person-time with BUP only without BZD/Z-drug	PD	Typical BZD/Z-drug supply: 30 days; typical BUP supply: 28 days (IQR 14–30 days)	Nonfatal drug-related poisoning (Opioid overdose, toxicity, naloxone reversal)	Administrative claims data	From January 1, 2006, to December 31, 2016 (up to 12 months after index poisoning event per patient)
Miano et al., 2022	Patient-time with BUP plus gabapentin, BUP plus clonidine, or BUP plus oxycodone	Person-time with BUP only without the interacting drugs	PD	Concurrent exposure periods: gabapentin 14–30 days, clonidine 7–30 days, oxycodone 3–7 days; 30 days per fill for chronic refills	Opioid overdose	*ICD*-coded overdose, ADE	From January 1, 2000, to December 31, 2019
Ellis et al., 2023	Patient-time with BUP plus gabapentin	Person-time with BUP only without gabapentin	PD	Gabapentin exposure defined as days covered by filled prescriptions within 90 days after initiation of BUP treatment; gaps > 45 days marked treatment discontinuation	Drug-related poisoning	*ICD*-coded poisoning, pharmacy claims	From January 1, 2006, to December 31, 2016,
Tardelli et al., 2023	Patient-time with BUP plus prescription amphetamine	Person-time with BUP only without prescription amphetamines	PD	Prescription-amphetamine exposure measured as overlapping 30-day intervals during BUP treatment, with follow-up up to 1 year after initiation	Drug-related poisoning	Administrative Claims data	From January 1, 2006, to December 31, 2016 (up to 12 months after index)
Schuman-Olivier et al., 2013	Patients with BUP plus BZD	Patients with BUP only without BZD	PD	Participants followed for 12 months from entry into office-based BUP treatment; BDZ exposure captured throughout the 1-year period via electronic medical records and urine screens.	ED visits	EMR, urine screens	From November 2007 to June 2010 (up to 12 months post OBOT intake)

Abbreviations: ADE, adverse drug event; AOR, adjusted odds ratio; AHR, adjusted hazard ratio; ARR, adjusted risk ratio; BUP, buprenorphine; BZD, benzodiazepine; CI, confidence interval; ED, emergency department; EMR, electronic medical record; HR, hazard ratio; *ICD*, *International Classification of Diseases*; IQR, interquartile range; NR, not reported; OBOT, office-based opioid treatment; OMT, opioid maintenance treatment; OUD, opioid use disorder; OR, odds ratio; PA, prescription amphetamine; PD, pharmacodynamic; PDMP, Prescription Drug Monitoring Program

**Table 3:** Summary of key findings by clinical outcomes and Interacting drug class

Clinical Outcome	Drugs Co-used With Buprenorphine	Direction of Effect	Key Findings	Standardized RR^a, b^
Fatal OD [26]	Benzodiazepines	Increased	aHR 2.92 (95% CI 2.10–4.06)	2.08
Non-fatal OD [26. 27]	Benzodiazepines	Mixed	aHR 2.05 (95% CI 1.68–2.44)	1.63
			aHR 1.05 (95% CI 0.51–2.15)	1.03
OD [27]	Z-drugs	Increased	aHR 4.38 (95% CI 2.09–9.17) ^c^	2.72
OD [27]	Pregabalin	Increased	aHR 2.84 (95% CI 1.48–5.45)	2.04
OD [28]	Gabapentin	Increased	aRR 2.17 (95% CI 1.09–4.31)	2.17
OD [28]	Clonidine	Increased	aRR 2.32 (95% CI 1.06–5.07)	2.32
OD [28]	Oxycodone	Increased	aRR 1.94 (95% CI 1.02–3.67)	1.94
Drug-related poisoning [29]	Benzodiazepines	Increased (dose-dependent)	Low-dose BZD: OR 1.11 (95% CI 1.00–1.23)	1.11
			High-dose BZD: OR 1.64 (95% CI 1.39–1.93)	1.64
Drug-related poisoning [30]	Gabapentin	Null	OR 0.98 (95% CI 0.85–1.13)	0.98
Drug-related poisoning [31]	Prescription amphetamines	Null	OR 0.96 (95% CI 0.75–1.23) in patients with co-occurring PSUD	0.96
			OR 0.96 (95% CI 0.84–1.10) in patients without co-occurring PSUD	0.96
ED visit [32]	Benzodiazepines	Increased	Injury-related ED visit: aOR 3.20 (95% CI 1.50–6.81)	3.20
			Psychiatric-related ED visit: aOR 1.86 (95% CI 0.87–3.99)	1.86
			Other medical ED visit: aOR 1.93 (95% CI 1.03–3.60)	1.93
			Overdose-related ED visit: null, aOR 1.19 (95% CI 0.23–6.22)	1.19
All-cause mortality [26,27]	Benzodiazepines	Mixed	aHR 1.90 (95% CI 1.48–2.44)	1.56
			aHR 1.44 (95% CI 0.93–2.23) ^d^	1.29
All-cause mortality [27]	Z-drugs	Increased	aHR 2.69 (95% CI 1.48–4.89)	1.97
All-cause mortality [27]	Pregabalin	Increased	aHR 1.82 (95% CI 1.10–3.01) ^d^	1.52

Abbreviations: aHR, adjusted hazard ratio; aOR, adjusted odds ratio; aRR, adjusted risk ratio; BUP, buprenorphine; BZD, benzodiazepine; DME, diazepam milligram equivalent; OD, overdose; OR, odds ratio; PSUD, psychostimulant use disorder. SUD, substance use disorder

^a^ HRs were converted to RR using a formula: (1 − 0.5^(√HR)^) ÷ (1 − 0.5^(√1/HR)^) by VanderWeele, with an assumption that the event rate is unknown but estimated between 0.2 and 0.8 for both study and comparison groups

^b^ ORs were converted to RR using a formula: ORs/[1-p+(p*ORs)] by VanderWeele, in which the event rate (P) in the non-exposed group is unknown, but estimated to be less than 10%

^c^ from the secondary analysis restricted to buprenorphine-only person-time (*n* = 3,221; 7,527 person-years) in the Abrahamsson et al. study

^d^ from the secondary analysis restricted to all OMT person-time (14,550 person-years) regardless of opioid maintenance treatment type in the Abrahamsson et al. study

### Opioid overdose

Three reviewed studies showed an increased risk of OD in patients or patient-times exposed to co-use of buprenorphine with benzodiazepines (aHR 2.92, 95% CI 2.10–4.06) [26], with Z-drugs (aHR 4.38, 95% CI 2.09–9.17), [27] with pregabalin (aHR 2.84, 95% CI 1.48–5.45) [27], with gabapentin (aRR 2.17, 95% CI 1.09–4.31) [28], with clonidine (aRR 2.32, 95% CI 1.06–5.07) [28], or with oxycodone (aRR 1.94, 95% CI 1.02–3.67) [28], compared with patients or patient-times exposed to buprenorphine only. Two studies showed mixed results on co-use of buprenorphine and benzodiazepines (vs. buprenorphine alone) and risk of nonfatal OD, with the U.S. study of 63,389 patients who initiated buprenorphine showing an increased risk (aHR 2.05, 95% CI 1.68–2.50) [26], and the Swedish study of 4,501 patients with opioid maintenance treatment indicated a null result for non-fatal OD (aHR 1.05, 95% CI 0.51–2.15) [27]. The standardized relative risks of fatal or non-fatal OD ranged between 2.72 and 1.03 across different types of drug class co-used with buprenorphine.

### Drug-related poisoning

A U.S. study of 23,036 patients with OUD and buprenorphine treatment showed a dose-response relationship between co-use of buprenorphine and benzodiazepines and increased risk of nonfatal drug-related poisoning (including opioid overdose) (OR 1.11, 95% CI 1.00–1.23 for low-dose [≤ 30 mg/day diazepam-equivalent] benzodiazepine; OR 1.64, 95% CI 1.39–1.93 for high-dose [(> 30 mg/day diazepam-equivalent] benzodiazepine) [29]. Two other U.S. studies showed null associations with risk of drug-related poisoning (including opioid overdose) in patients or patient-times exposed to co-use of buprenorphine with gabapentin (OR 0.98, 95% CI 0.85–1.13) [30] or with prescription amphetamines (OR 0.96, 95% CI 0.75–1.23 and OR 0.96, 95% CI 0.84–1.10 in a subgroup with or without, respectively, polysubstance use disorder) [31]. The standardized relative risks of drug-related poisoning ranged between 0.96 and 1.64 across different types of drug classes co-used with buprenorphine.

### ED visits

A study of 328 patients who received office-based buprenorphine treatment in a single U.S. clinical center showed that co-use of buprenorphine and benzodiazepines (vs. buprenorphine alone) was associated with increased odds of injury-related ED visits (aOR 3.20, 95% CI 1.50–6.81), psychiatric-related ED visits (aOR 1.86, 95% CI 0.87–3.99), and other medical ED visits (aOR 1.93, 95% CI 1.03–3.60), but not associated with odds of overdose-related ED visits (aOR 1.19, 95% CI 0.23–6.22) [32]. The standardized relative risks of different types of ED visits ranged between 0.96 and 1.64 in the co-use of buprenorphine and benzodiazepine.

### All-cause mortality

A Swedish study of 4,501 patients with opioid maintenance treatment showed an increased risk of all-cause mortality in patients who co-used buprenorphine with Z-drugs (aHR 2.69, 95% CI 1.48–4.89) or with pregabalin (aHR 1.82, 95% CI 1.10–3.01), compared with buprenorphine only [27]. Two studies showed mixed results on co-use of buprenorphine with benzodiazepines vs. buprenorphine only, with the U.S. study of 63,389 patients who initiated buprenorphine showing an increased risk (aHR 1.90, 95% CI 1.48–2.44) [26], and the Swedish study of 4,501 patients indicating a null result for all-cause mortality (aHR 1.44, 95% CI 0.93–2.23) [27]. The standardized relative risks of all-cause mortality ranged between 1.29 and 1.97 across different types of drug classes co-used with buprenorphine.

## Discussion

This systematic review is among the first to synthesize current evidence on the associations of buprenorphine–drug interactions with clinical outcomes. All seven studies identified were observational with no RCT. These observational studies had heterogeneity in the studied drug (including benzodiazepines, Z-drugs, gabapentinoids [gabapentin and pregabalin], clonidine, oxycodone, and prescription amphetamines) that potentially interact with buprenorphine, and heterogeneity in outcomes of interest (including opioid overdose, drug-related poisoning, all-cause ED visits, and all-cause mortality), which prevented us from conducting a meta-analysis. The review showed increased odds of drug-related poisoning (including opioid overdose) or ED visits associated with concomitant buprenorphine–benzodiazepine use, with mixed associations for opioid overdose and all-cause mortality among patients with OUD. In addition, co-use of buprenorphine with Z-drugs or pregabalin was associated with increased risk of opioid overdose and all-cause mortality, but associations of co-use with drug-related poisoning or ED visits were null. Overall, this review of seven observational studies suggests some, but not conclusive, evidence on potential adverse outcomes associated with concomitant use of buprenorphine and other drugs when compared with buprenorphine use only among patients with OUD.

The inconclusive evidence on the association between buprenorphine–drug interactions and risk of clinical outcomes among patients with OUD may be explained by differences in the data sources (commercial insurance claims or electronic health data vs. registry data), differences in health system, study design (cohort vs. case-only study), and study sample (new vs. prevalent users of buprenorphine), as well as differences in the types of the studied buprenorphine–drug interactions and outcomes. All seven studies evaluated PD interactions between buprenorphine and medications with potential CNS depressant properties. The mechanism of the PD interaction is that the combined use of buprenorphine and these CNS depressants would synergistically enhance the level of sedation and respiratory depression, leading to risk of overdose, cerebral hypoxia (which is brain damage from lack of oxygen), and death [15, 16]. The risk of adverse clinical outcomes may be heightened by buprenorphine, indicated for OUD vs. pain, particularly given the differences in dosing practices. A once-daily dose of buprenorphine (8–24 milligrams after induction) is recommended for OUD to suppress opioid withdrawal symptoms that last between 24 and 36 h, whereas a low or divided dose of buprenorphine is prescribed and administered two to three times daily because the drug’s analgesic effect lasts approximately 4 to 8 h [3].

Several methodological limitations were observed in existing studies. Chief among these limitations is comparing persons or person-times with concurrent buprenorphine–drug exposure vs. those with no active treatment (i.e., buprenorphine only), which leads to confounding by indication. Individuals who had co-prescribed buprenorphine and CSN depressants, such as benzodiazepines, often have OUD and comorbid psychiatric conditions, strong risk factors of opioid overdose and mortality [33, 34]. Failure to compare patients receiving alternative treatments for similar conditions in prior studies [26–32] limits their ability to understand whether the risk of clinical outcomes is associated solely with concurrent use of a specific combination of buprenorphine–drug interaction. More population-based studies with active comparators are needed to provide robust evidence regarding associations between buprenorphine–drug interactions and clinical outcomes.

The existing studies are also limited to examining buprenorphine–drug interactions based on PD interactions. None of the studies assessed PK interactions between buprenorphine and CYP3A4 modulators. Buprenorphine is primarily metabolized by the CYP3A4 enzyme, and co-use of buprenorphine with drugs that inhibit or induce the CYP3A4 enzyme can increase or reduce buprenorphine metabolism, affecting buprenorphine efficacy and triggering withdrawal [13]. Clinical trials show that voriconazole, a CYP3A4 inhibitor, nearly doubles buprenorphine exposure, whereas rifampin, a potent CYP3A4 inducer, significantly lowers plasma levels of buprenorphine, causing withdrawal in some patients [14, 35]. Despite these known PK mechanisms, no observational studies have quantified population-based clinical outcomes associated with such interactions. This gap may leave clinicians uncertain about the risks of altered buprenorphine metabolism in practice. Future investigations that integrate PK mechanisms with pharmacoepidemiologic methodology may help elucidate clinical outcomes associated with PK interactions involving buprenorphine [28].

Another notable limitation is that existing studies investigated buprenorphine–drug interactions among patients with OUD only, with no studies focusing on patients who received buprenorphine for chronic pain. While frequently prescribed for OUD, buprenorphine is increasingly considered by U.S. clinicians as a viable opioid treatment or replacement for full agonist opioids (e.g., hydrocodone) for managing pain that is related to cancer or has non-cancer origins [8, 9], given its safety profile and less federally regulated status as a Schedule III controlled substance, compared with conventional full-opioid agonists, most of which are Schedule II substances [36]. In the 2022 Updated U.S. Departments of Veterans Affairs (VA) and Defense (DoD) Clinical Practice Guideline, buprenorphine was first recommended as the first-line treatment to replace full agonist opioids for patients who require daily opioids for pain management, despite scarce data supporting such a recommendation [37]. While specific to the VA/DoD, this guideline, based on limited evidence, may expand into the greater U.S. health care system, given the growing population of patients with chronic pain who require opioid therapy. Several clinical issues remain unanswered on buprenorphine prescribing, including buprenorphine–drug interactions and unintended outcomes. Future studies in this area are needed.

An additional limitation is that all studies included in the review were observational, and thus the results were subject to residual confounding, exposure misclassification, and unmeasured polysubstance use. These reviewed studies have considerable heterogeneity in outcomes of interest, signaling a need to standardize outcomes in future observational studies. Publication bias could not be assessed due to the small evidence base. Collectively, these findings should be viewed as safety signals rather than causal estimates, highlighting the need for well-controlled, active comparator studies integrating pharmacokinetic and pharmacoepidemiologic methods.

While requiring further studies to address the limitations, the current evidence may provide clinical implications regarding the potential risk of adverse outcomes associated with concurrent use of buprenorphine and studied drugs, all of which have CNS depressant effects. When buprenorphine is used to manage OUD, clinical attention is particularly needed for patients who also receive medications with CNS depressant properties. It may also be important to educate patients about the potential interactions of buprenorphine and CNS depressants and the importance of avoiding other CNS sedatives while receiving buprenorphine treatment [34]. Overall, balancing the potential benefits of buprenorphine and the CNS depressants associated with increased risk of poor outcomes (e.g. benzodiazepines, pregabalin) and the potential risks is essential to manage individual clinical need and achieve treatment goals in patients with OUD [38].

## Conclusion

This review provides synthesized evidence on clinical outcomes associated with co-prescribing buprenorphine and CNS depressants. There is some, but not conclusive, evidence on potential adverse outcomes, including opioid overdose, drug-related poisoning (including opioid overdose), all-cause ED visits, and all-cause mortality, associated with different combinations of concomitant buprenorphine and CNS depressants, when compared with prescribing buprenorphine alone. Associations of concomitant buprenorphine–drug use with risk of clinical outcomes merit further investigation in population-based studies with active comparator designs and considerations of pharmacokinetic buprenorphine–drug interactions and patients with chronic pain treated with buprenorphine.

## Supplementary Information

Below is the link to the electronic supplementary material.

Supplementary Material 1

Supplementary Material 2

Supplementary Material 3

Supplementary Material 4

## Data Availability

All data analyzed in this study were derived from published sources cited in the reference list.
