# Supplementary material for: Clinical outcomes associated with drug–drug interactions involving buprenorphine: a systematic review
Source: Addict Sci Clin Pract. 2026 Jul 9;21:70. doi: 10.1186/s13722-026-00698-8 (PMC13637234; doi:10.1186/s13722-026-00698-8)
Supplement: Supplementary file 1 [file 13722_2026_698_MOESM1_ESM.docx]

**Supplementary Material: Appendix S1. Eligibility Criteria**

| **Domain** | **Inclusion Criteria** | **Exclusion Criteria** |
| --- | --- | --- |
| Databases and Platforms | MEDLINE with Full Text (EBSCOhost, 1946–present), CINAHL Plus with Full Text (EBSCOhost, 1937–present), APA PsycINFO (EBSCOhost, 1806–present), and Web of Science Core Collection (Clarivate, 1900–present). Searches conducted June 16, 2025. | Grey literature sources, unindexed repositories, and databases outside the stated platforms. |
| Search Coverage and Yield | Combined search retrieved 1,578 records (527 MEDLINE; 513 CINAHL; 317 PsycINFO; 221 Web of Science). Search strings included controlled vocabulary (MeSH, CINAHL Headings) and keyword fields (TX, XB, AB, TI). | Studies not captured in these searches or lacking abstracts in English. |
| Study Design | Randomized clinical trials (RCTs) or observational studies (cohort, case-control, case-crossover, or self-controlled case series) evaluating real-world buprenorphine drug interactions. | Qualitative or review articles, case reports, animal or in vitro studies, and forensic toxicology reports without clinical outcomes. |
| Population | Adults (≥18 years of age) receiving buprenorphine for opioid use disorder, opioid dependence, or chronic pain | Studies limited to methadone, naltrexone, or other opioid agonists without buprenorphine exposure, or pediatric samples. |
| Exposure/Interacting Drugs | Concurrent (co-use or co-prescription) of buprenorphine with: (a) central nervous system depressants (benzodiazepine, Z-drug, gabapentinoid, clonidine, prescription amphetamine, opioid analgesic) or (b) CYP3A4 modulator (inhibitor or inducer). | Buprenorphine monotherapy only or studies not evaluating co-use with another drug. |
| Comparator | Patient-time exposed to buprenorphine alone or unexposed reference periods within the same individuals. | Studies without a defined comparison group or reference exposure period. |
| Outcomes | Opioid poisoning, drug-related poisoning, opioid overdose, opioid withdrawal symptoms, respiratory depression, CNS depression, hospitalization, emergency department visits, adverse drug reaction, harm, or death. | Studies reporting only laboratory parameters, PK simulations without clinical endpoints, or non-clinical outcomes. |
| Setting and Data Source | RCT or observational studies using real-world data sources, such as electronic health records, claims databases, prescription monitoring programs, or national registries. | Experimental pharmacology settings, non-treatment populations, or non-clinical laboratory studies. |
| Language and Publication Status | English-language, peer-reviewed articles published through 2025. | Non-English publications, preprints, conference abstracts, or unpublished dissertations without peer review. |
| Special Note on CYP3A4 Studies | PK interaction studies evaluating CYP3A4 inducers or inhibitors were eligible; however, no studies meeting these criteria were identified after full-text screening. |  |

Abbreviations: PK, pharmacokinetic; CNS, Central Nervous System; RCT, Randomized Controlled Trial
