# Supplementary material for: Clinical outcomes associated with drug–drug interactions involving buprenorphine: a systematic review
Source: Addict Sci Clin Pract. 2026 Jul 9;21:70. doi: 10.1186/s13722-026-00698-8 (PMC13637234; doi:10.1186/s13722-026-00698-8)
Supplement: Supplementary file 2 [file 13722_2026_698_MOESM2_ESM.docx]

**Supplementary Material: Appendix S3. Search Queries**

| Database Name | Platform | Date Coverage | Date of Search | Number of studies pooled |
| --- | --- | --- | --- | --- |
| Medline | EBSCOHOST | 1946–present | 6/16/2025 | 527 |
| CINAHL | EBSCOhost | 1937–present | 6/16/2025 | 513 |
| APA PsycINFO | EBSCOhost | 1806–present | 6/16/2025 | 317 |
| Web of Science | Clarivate | 1900–present | 6/16/2025 | [221](https://www-webofscience-com.proxy.lib.ohio-state.edu/wos/woscc/summary/ad371c04-4430-47b7-b0a7-f54e61875c8e-01689d979c/relevance/1) |

|  |  | **MEDLINE** | **CINAHL** | **APA PsycINFO** |
| --- | --- | --- | --- | --- |
| Steps | Search string | Number of results | Number of results | Number of results |
| 1. Population terms | TX (‘Opioid-Related Disorders’ OR “substance-related disorders" OR “OUD" OR "medication assisted treatment" OR “medication-assisted treatment" OR "MAT" OR "MOUD" OR “opioid agonist therapy” OR “opioid agonist treatment” OR "maintenance therapy") | 254,081 | 53,845 | 46,006 |
| 2. | XB (opioid* OR opiat* OR opioid* OR opiat*) | 85,572 | 40,437 | 51,397 |
| 3. | XB (abuse* OR addict* OR depend* OR disorder*) | 2,182,344 | 1,084,033 | 648,289 |
| 4. | #2 AND #3 | 32,144 | 23,471 | 18,760 |
| 5. | MH ("chronic pain" OR "pain management" OR "neuropathic pain" OR "cancer pain" OR "low back pain" OR "fibromyalgia" OR  "noncancer pain" OR "persistent pain") | 104,511 | 10,938 | 75,428 |
| 6 | #1 OR #4 OR #5 | 366,502 | 78,215 | 129,423 |
| 7. Intervention | XB (buprenorphine OR Suboxone OR Suboxone OR Subutex OR Subutex) | 10,596 | 9,831 | 8,911 |
| 8. | #6 AND #7 | 6,527 | 5,536 | 5,023 |
| 9. Interacting drugs | All fields (benzodiazepine* OR gabapentin* OR pregabalin OR clonidine OR guanfacine OR Cannab* OR stimulant* OR Amphetamine* OR methamphetamine* OR ecstasy OR Psychostimulant*OR fluoxetine OR paroxetine OR sertraline OR venlafaxine OR duloxetine OR Antidepressant* OR Antipsychotic* OR carbamazepine OR phenytoin OR valproate OR lamotrigine OR topiramate OR Anticonvulsant* OR ritonavir OR efavirenz OR Atazanavir OR Delavirdine OR antiretroviral* OR cobicistat OR ketoconazole OR itraconazole OR clarithromycin OR erythromycin OR rifampin OR St. John’s wort OR sofosbuvir OR ledipasvir OR velpatasvir OR ombitasvir OR elbasvir OR grazoprevir OR glecaprevir OR pibrentasvir OR ribavirin OR DAA OR direct-acting antiviral* OR hepatitis C treatment) | 715,460 | 193,822 | 155,363 |
| 10. Outcomes | All fields ("Opioid poisoning" OR "drug-related poisoning" OR "opioid overdose" OR "opioid withdrawal symptoms" OR “withdrawal” OR "CNS depression" OR "respiratory depression" OR "hospitalization" OR "emergency department visit*" OR “ED visit*” OR "adverse drug reaction*" OR "harm*" OR "death" | 1,922,410 | 350,090 | 472,676 |
| 11. Drug-drug interaction | "CYP enzyme" OR "drug-drug interactions" OR “drug interactions" OR “DDI” OR 'cytochrome p450 enzyme' OR 'cyp p450' OR  'cyp3a4' OR “pharmacodynamic” OR “pharmacokinetic”) | 291,481 | 1,733 | 18,140 |
| Final combined query | #8 AND #9 AND #10 and #11 | 527 | 513 | 317 |

KEY:

XB = TITLE AND ABSTRACT

MH = MAJOR EXACT SUBJECT HEADING

TX = ALL TEXT

**Web of Science Core Collection**

| Steps | Search string | Number of results |
| --- | --- | --- |
| 1. Population terms | AB = (‘Opioid-Related Disorders’ OR “substance-related disorders" OR “OUD" OR "medication assisted treatment" OR "MAT" OR "MOUD" OR "maintenance therapy") | [52,538](https://www-webofscience-com.proxy.lib.ohio-state.edu/wos/woscc/summary/abee401b-5066-4221-b83b-782bffe5b92b-01689d3cc9/relevance/1) |
| 2. | AB = (opioid* OR opiat* OR opioid* OR opiat*)) | [83,261](https://www-webofscience-com.proxy.lib.ohio-state.edu/wos/woscc/summary/3c16c00c-3145-44e1-af83-e7665054677b-01689d475c/relevance/1) |
| 3. | AB=((abuse* OR addict* OR depend* OR disorder*) | [1,454,141](https://www-webofscience-com.proxy.lib.ohio-state.edu/wos/woscc/summary/9ac7f707-e931-4d53-afcb-b3374984cd39-01689d48b3/relevance/1) |
| 4. | #2 AND #3 | [14,641](https://www-webofscience-com.proxy.lib.ohio-state.edu/wos/woscc/summary/9984b4cc-d31f-481a-9f8a-0da3828b1d11-01689d61c2/relevance/1) |
| 5. | AB=(("chronic pain" OR "pain management" OR "neuropathic pain" OR "cancer pain" OR "low back pain" OR "fibromyalgia" OR "noncancer pain" OR "persistent pain")) | [143,506](https://www-webofscience-com.proxy.lib.ohio-state.edu/wos/woscc/summary/32ab0707-b210-4ed6-b571-50eefc57f09d-01689cfc2c/relevance/1) |
| 6. | #1 OR #4 OR #5 | [205,880](https://www-webofscience-com.proxy.lib.ohio-state.edu/wos/woscc/summary/4c764367-0e59-4807-9be7-2575ea3246c7-01689d8fc5/relevance/1) |
| 7. Intervention | AB=(buprenorphine OR naloxone OR Suboxone OR Suboxone OR Subutex OR Subutex) | [30,599](https://www-webofscience-com.proxy.lib.ohio-state.edu/wos/woscc/summary/e70442e9-9717-4d82-b28d-4b94e85c5edd-01689cfb54/relevance/1) |
| 8. | #6 AND #7 | [5,644](https://www-webofscience-com.proxy.lib.ohio-state.edu/wos/woscc/summary/6b4e8c54-bf25-4821-8c93-8dec376e5d3d-01689d931d/relevance/1) |
| 9. Interacting Drugs   i) CNS and neuropsychiatric drugs | AB = (benzodiazepine* OR gabapentin* OR pregabalin OR clonidine OR guanfacine OR Cannab* OR stimulant* OR Amphetamine* OR methamphetamine* OR ecstasy OR Psychostimulant*OR fluoxetine OR paroxetine OR sertraline OR venlafaxine OR duloxetine OR Antidepressant* OR Antipsychotic* OR carbamazepine OR phenytoin OR valproate OR lamotrigine OR topiramate OR Anticonvulsant* ) | [318,668](https://www-webofscience-com.proxy.lib.ohio-state.edu/wos/woscc/summary/0ee1fed2-7a82-4061-8b85-cf296d70ae6f-01689cfa12/relevance/1) |
| 10. Interacting Drugs   i) Anti-infective and hepatitis C drugs | AB = (ritonavir OR efavirenz OR Atazanavir OR Delavirdine OR antiretroviral* OR cobicistat OR ketoconazole OR itraconazole OR clarithromycin OR erythromycin OR rifampin OR St. John’s wort OR sofosbuvir OR ledipasvir OR velpatasvir OR ombitasvir OR elbasvir OR grazoprevir OR glecaprevir OR pibrentasvir OR ribavirin OR DAA OR direct-acting antiviral* OR hepatitis C treatment) | [177,051](https://www-webofscience-com.proxy.lib.ohio-state.edu/wos/woscc/summary/42c25937-4404-450f-a611-b3ec8c350e91-01689cf993/relevance/1) |
| 11. | #9 OR #10 | [493,038](https://www-webofscience-com.proxy.lib.ohio-state.edu/wos/woscc/summary/97b63596-2a04-4da0-ba65-202c5016e8f2-01689d1bee/relevance/1) |
| 12.a Clinical outcomes | AB = ("Opioid poisoning" OR "drug-related poisoning" OR "opioid overdose" OR "opioid withdrawal symptoms" OR “withdrawal” OR "CNS depression" OR "respiratory depression") | [184,591](https://www-webofscience-com.proxy.lib.ohio-state.edu/wos/woscc/summary/8cb69dff-2236-4fc9-b5f2-3e105935153c-01689cf79a/relevance/1) |
| 12.b Clinical outcomes | TI =("hospitalization" OR "emergency department visit*" OR “ED visit*” OR "adverse drug reaction*" OR "harm*" OR "death" ) | [422,501](https://www-webofscience-com.proxy.lib.ohio-state.edu/wos/woscc/summary/b04e9297-09f7-4745-b5bf-e3a626808a56-01689d75cb/relevance/1) |
| 13. Drug-drug interaction | TI = "CYP enzyme" OR "drug-drug interactions" OR “drug interactions" OR “DDI” OR 'cytochrome p450 enzyme' OR ‘CYP enzyme’ OR 'cyp3a4' OR “pharmacodynamic interaction.” | 15,597 |
| 14 | #12.a OR #12.b OR #13 | [620,470](https://www-webofscience-com.proxy.lib.ohio-state.edu/wos/woscc/summary/979b5151-dfd7-4c42-8893-47523a0e5779-01689d851d/relevance/1) |
| Final combined query | #8 AND #11 AND #14 | [221](https://www-webofscience-com.proxy.lib.ohio-state.edu/wos/woscc/summary/ad371c04-4430-47b7-b0a7-f54e61875c8e-01689d979c/relevance/1) |

KEY:

AB = ABSTRACT

TI = TITLE SEARCH
