# Supplementary material for: Clinical outcomes associated with drug–drug interactions involving buprenorphine: a systematic review
Source: Addict Sci Clin Pract. 2026 Jul 9;21:70. doi: 10.1186/s13722-026-00698-8 (PMC13637234; doi:10.1186/s13722-026-00698-8)
Supplement: Supplementary file 3 [file 13722_2026_698_MOESM3_ESM.docx]

**Supplementary Material: Appendix S4. Quality Assessments**

**Newcastle Ottawa Scale Quality Assessment [1]**

| **Study** | **S1** | **S2** | **S3** | **S4** | **C1** | **C2** | **O1** | **O2** | **Total (0–9)** | **Overall Quality** |
| --- | --- | --- | --- | --- | --- | --- | --- | --- | --- | --- |
| **Abrahamsson et al., 2017** |  |  |  |  |  | **🔴** |  |  | **8** | **High** |
| **Park et al., 2020** |  |  |  |  |  |  |  |  | **9** | **High** |
| **Schuman-Olivier et al., 2013** |  |  |  |  |  | **🔴** |  |  | **8** | **High** |

*Studies lacking an active pharmacologic comparator (e.g., exposed vs unexposed design) were penalized one star under the Comparability (C2) domain unless robust multivariable or time-varying adjustment effectively minimized confounding by indication. Park et al. (2020) retained full comparability points due to extensive adjustment, while Abrahamsson et al. (2017) and Schuman-Olivier et al. (2013) each lost one point for the absence of an active comparator*.

Scale:

High quality: 7–9 stars

Moderate quality: 4–6

Low quality: 0–3

Domain assessed:

S1 = Representativeness of the exposed cohort: whether the exposed participants accurately reflect the average individuals in the target population.
S2 = Selection of the non-exposed cohort: whether the comparison group was drawn from the same source population as the exposed group.
S3 = Ascertainment of exposure: how accurately exposure (e.g., prescription or treatment) was identified, preferably through validated or secure records.
S4 = Outcome not present at start: confirmation that participants were outcome-free at baseline before follow-up.
C1 = Control for key confounders: whether analyses adjusted for the most important confounding variables (e.g., age, sex, psychiatric comorbidities).
C2 = Control for additional factors/use of active comparator: whether further confounders were controlled or an active comparator was used to reduce confounding by indication.
O1 = Assessment of outcome: whether outcomes were ascertained using reliable, independent, or validated sources (e.g., medical records, registries).
O2 = Length of follow-up: whether the duration of observation was sufficient for the outcome to occur.
O3 = Adequacy of follow-up: whether follow-up was complete and loss to follow-up was minimal or unlikely to bias results.

**NOS Quality Assessment adapted from Wachira et al., 2022 [2]**

| **Study** | **S1** | **S2** | **S3** | **C1** | **C2** | **O1** | **O2** | **O3** | **O4** | **O5** | **Total (0–10)** | **Overall Quality** |
| --- | --- | --- | --- | --- | --- | --- | --- | --- | --- | --- | --- | --- |
| **Xu et al., 2021** |  |  |  |  |  |  |  |  |  |  | **10** | **High** |
| **Tardelli et al., 2023** |  |  |  |  | **🔴** |  |  |  |  |  | **9** | **High** |
| **Ellis et al., 2023** |  |  |  |  | **🔴** |  |  |  |  | **🔴** | **8** | **Moderate–High** |
| **Miano et al., 2022** |  |  |  |  |  |  |  |  |  |  | **10** | **High** |

*All studies used validated data sources, defined risk/control intervals, and appropriately handled time-varying confounders. Xu et al. (2021) and Miano et al. (2022) achieved full marks, while Ellis et al. (2023) lacked follow-up detail, and Tardelli et al. (2023) did not explicitly adjust for all temporal confounders. Overall, all studies were rated high or moderate–high quality.*

Domain assessed:

S1 = Representativeness of cases:
Evaluates whether the included cases accurately represent all eligible individuals from the source population. A study earns a star if cases are drawn from a comprehensive, well-defined population (e.g., national surveillance, administrative registry) rather than a selected subgroup or convenience sample.

S2 = Ascertainment of exposure:
Assesses the reliability of exposure measurement. A star is awarded when exposure is verified using a secure or validated data source such as prescription or vaccination registries, pharmacy claims, or electronic medical records, rather than unverified self-report.

S3 = Outcome not present at start:
Checks that the outcome of interest did not exist at the beginning of the observation period, ensuring that events occurred after the defined exposure or within the risk window. This confirms correct temporal sequencing between exposure and outcome.

C1 = Adjustment for time-varying confounders (e.g., age, seasonality):
Examines whether the study design or analysis controlled for temporal factors that may vary within individuals and bias associations such as age effects, seasonality, or calendar time trends through statistical adjustment or by limiting follow-up intervals.

C2 = Adjustment for additional confounders or justification of limited control:
Determines whether other relevant covariates (e.g., medication changes, comorbidities) were addressed, or whether the authors provided a justified rationale for not doing so. A star is awarded when additional confounding was considered, or the control strategy is well-justified.

O1 = Assessment of outcome:
Evaluates how outcomes were measured and verified. High-quality studies use objective or validated ascertainment methods such as ICD-coded diagnoses, laboratory confirmation, or independent clinical assessment rather than self-report alone.

O2 = Risk period clearly stated:
Confirms that the time window of increased risk following exposure (e.g., 0–30 days post-exposure) was explicitly defined and justified based on prior literature or biological plausibility.

O3 = Control period clearly stated:
Ensures that a distinct, well-described comparison period (before, after, or between risk intervals) was defined to estimate background incidence, allowing within-person comparison of event rates.

O4 = Risk/control periods long enough for outcome to occur:
Assesses whether the observation windows were sufficient to capture the onset of the event of interest, with rationale provided for the chosen durations (e.g., 30-day or 90-day risk periods).

O5 = Adequacy of follow-up:
Evaluates completeness of case observation throughout the study. Full credit is given when all cases are accounted for or when losses are small, well-described, and unlikely to bias results (e.g., deaths or censored records clearly handled)

Scale:

High quality: 7–10 stars

Moderate quality: 4–6 stars

Low quality: ≤3 stars

References

1. Wells, G. A., Shea, B., O’Connell, D., Peterson, J., Welch, V., Losos, M., & Tugwell, P. (2014). The Newcastle–Ottawa Scale (NOS) for assessing the quality of nonrandomised studies in meta-analyses. Ottawa Hospital Research Institute. http://www.ohri.ca/programs/clinical_epidemiology/oxford.asp
2. Virginia KW, Henry MP, Maria Regina FO (2022) Proposal of a Quality Assessment Tool for the Evaluation of the Methodological Quality of Self-controlled Case Series and Self-controlled Risk Interval Study Designs. J Community Med Health Educ 12:770. DOI: 10.4172/2168-9717.1000770
