# Supplementary material for: Clinical outcomes associated with drug–drug interactions involving buprenorphine: a systematic review
Source: Addict Sci Clin Pract. 2026 Jul 9;21:70. doi: 10.1186/s13722-026-00698-8 (PMC13637234; doi:10.1186/s13722-026-00698-8)
Supplement: Supplementary file 4 [file 13722_2026_698_MOESM4_ESM.docx]

**Supplementary Material: Appendix S2, Definition of Outcomes**

| **Outcomes** | **Definition** |
| --- | --- |
| Opioid poisoning | Self-reported or biologically tested exposure to opioid poisoning or having a diagnosis of opioid poisoning based on International Classification of Diseases (ICD)-9 or ICD-10 codes, recorded in patient disease registry, administrative claims, or electronic health data.  ICD-9 codes: 965, E850  ICD-10 codes: T40 |
| Drug-related poisoning | Self-reported or biologically tested exposure to drug-related poisoning or having a diagnosis of drug-related poisoning based on International Classification of Diseases (ICD)-9 or ICD-10 codes, recorded in patient disease registry, administrative claims, or electronic health data.  ICD-9 codes: 960, 961, 962, 963, 964, 965, 966, 967, 968, 969, 970, 971, 972, 973,974, 975,976,977,978, 979, 980, 981,982, 983, 984, 985, 986, 987,988, 989, E850, E851, E852, E853, E854, E855, E856, E857, E858, E860, E861, E862, E863, E864, E865, E866, E867, E868, E869, E950, E951, E952, E962, E972, E975, E976, E980, E981, E982  ICD-10 codes: T40, T41, T42, T43, T44, T45, T46, T47, T48, T49, T50, T51, T52, T53, T54, T55, T56, T57, T58, T59, T60, T61,T62, T63, T64, T65  Drug-related poisoning encompasses poisoning by opioids, benzodiazepines and sedatives, psychotropic agents, CNS stimulants, cardiovascular agents, and other substances, and therefore includes but is not limited to opioid poisoning. The specific ICD codes applied may vary across the reviewed studies. |
| Opioid overdose | Self-reported or biologically tested exposure to opioid poisoning or having a diagnosis of opioid overdose based on International Classification of Diseases (ICD)-9 or ICD-10 codes, recorded in patient disease registry, administrative claims, or electronic health data.  ICD-9 codes: 965, E850  ICD-10 codes: T40 |
| Opioid withdrawal symptoms | Opioid withdrawal symptoms were assessed using the Clinical Opiate Withdrawal Scale (COWS) or Subjective Opiate Withdrawal Scale (SOWS) documented in a disease registry or electronic health data. The COWS score of 5-12 indicates mild symptoms, 13-24, moderate symptoms, 25-36, moderately severe symptoms, and more than 36, severe withdrawal symptoms. The SOWs score of 1-10 indicates mild withdrawal, 11-20, moderate withdrawal, 21-30, and severe withdrawal. |
| Respiratory depression | Having an event of respiratory depression recorded in a disease registry or electronic health data |
| Central Nervous System depression | Having an event of CNS depression recorded in a disease registry or electronic health data |
| Hospitalization | Self-reported hospitalization or having an inpatient encounter recorded in patients’ disease registry, administrative claims, or electronic health data |
| Emergency department visit | Self-reported hospitalization or having a medical encounter at an emergency department, recorded in a disease registry, administrative claims, or electronic health data |
| Adverse drug reaction | Self-reported adverse drug reaction documented in patient disease registry or electronic health data |
| Harm | Self-reported harm (s) documented in patient disease registry or electronic health data |
| Death | Death event documented in patient disease registry, administrative claims data, or electronic health data |
